# Coronary Computed Tomography Angiography Solving Ambiguity in Chronic Total Occlusion Percutaneous Coronary Intervention

**DOI:** 10.1016/j.jscai.2023.101261

**Published:** 2024-03-26

**Authors:** Nikoloz Shekiladze, Hiroki Ueyama, Pratik Sandesara, Nodari Maisuradze, Patrick Gleason, William J. Nicholson

**Affiliations:** a Section of Interventional Cardiology, Division of Cardiovascular Medicine, Emory University School of Medicine, Atlanta, Georgia; b Internal Medicine, State University of New York Downstate Medical Center, Brooklyn, New York

**Keywords:** coronary computed tomography angiography, chronic total occlusion, percutaneous coronary intervention

Although success rate for chronic total occlusion (CTO) percutaneous coronary interventions (PCI) have increased in recent years, outcomes remain highly variable depending on operator experience and anatomic characteristics (ie, proximal cap ambiguity, long occlusion length, vessel tortuosity and calcification).^1^ Coronary computed tomography angiography (CCTA) has been established in preprocedural planning of CTO PCI as it can reliably depict proximal cap morphology, calcifications, and tortuosity within the CTO segment and occlusion length.^2^ Pre-procedural CCTA guidance for CTO PCI is associated with higher success rates and fewer periprocedural complications,^3^ and therefore an essential modality to learn. We present cases where CCTA played an instrumental role: case 1, elucidating proximal cap morphology in relation to side branch arteries in left anterior descending artery CTO (Figure 1A); case 2, delineating the trajectory of an anomalous left circumflex coronary artery CTO originating from proximal right coronary artery (RCA) (Figure 1B); and case 3, identifying tandem CTO lesions in the RCA (Figure 1C).

**Figure 1:**
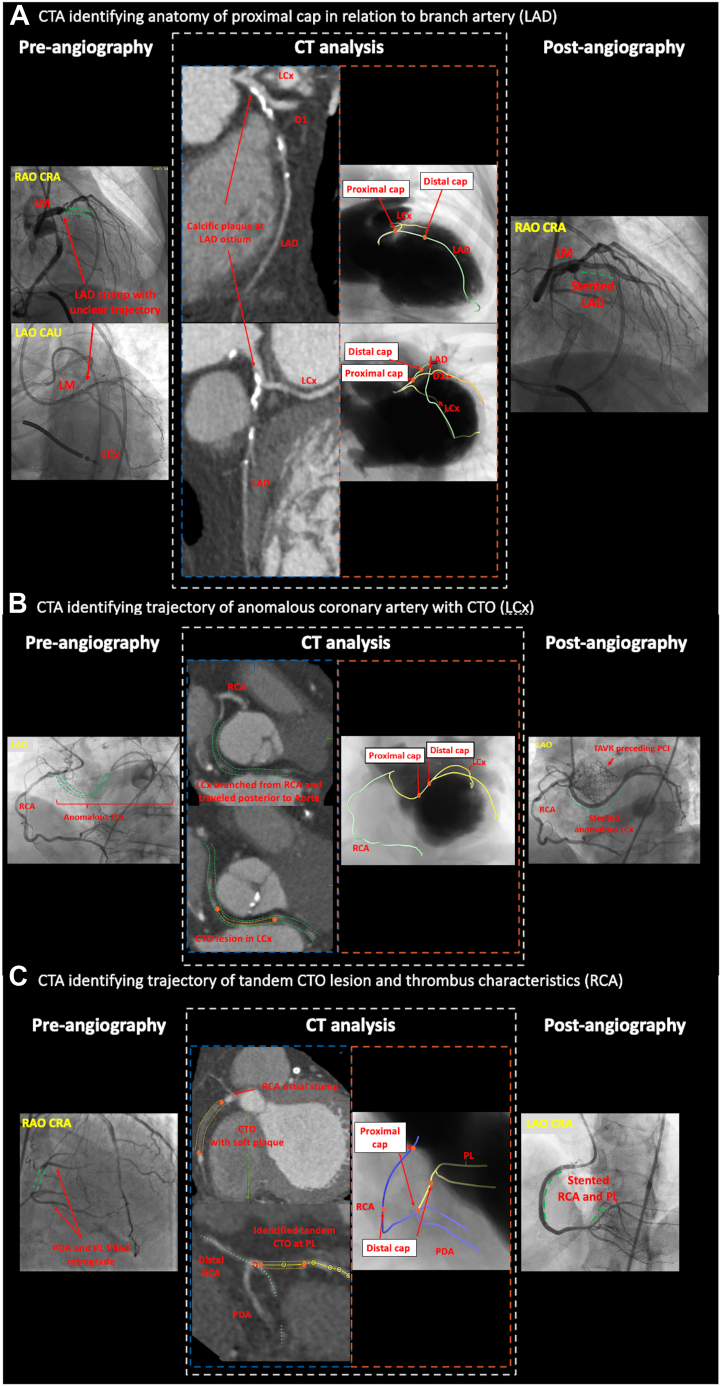
**CT pre-planning of the cases and angiographic outcomes**. (**A**) Diagnostic angiography showed LAD stump with unclear trajectory (Supplemental Video 1). CCTA-curved MPR images identified proximal cap morphology and ostial LAD take-off in relation to D1 and LCx. Coregistered roadmap created to delineate ambiguous LAD CTO take-off. LAD trajectory is delineated as green, LCx as yellow, and D1 as orange. Interrupted green line represents CTO lesion in the LAD before and after stenting. (**B**) Diagnostic angiography showed retrograde filling of anomalous LCx CTO originating from RCA (Supplemental Video 2). CCTA MPR images identified fibrotic morphology of the proximal/distal cap and retroaortic course of the anomalous LCx. Coregistered roadmap visualized the length of occlusion. RCA trajectory is delineated as green and anomalous LCx as yellow. Interrupted green line represents trajectory of anomalous LCx before and after stenting. Interrupted orange line represents the CTO lesion. (**C**) Diagnostic angiography showed RCA ostial CTO with unclear trajectory and retrograde filling of PDA and PL (Supplemental Video 3). CCTA MPR images identified fibrotic morphology of proximal/distal caps and tandem CTO lesions. Coregistered roadmap created to delineate tandem lesion and guide angle of approach. RCA and PDA trajectory are delineated as blue and PL as yellow. Interrupted green line represents trajectory of RCA and PDA before and after stenting. Interrupted orange line represents the CTO lesions. CCTA, computed coronary tomographic angiography; CTO, chronic total occlusion; D1, first diagonal; LAD, left anterior descending; LCx, left circumflex; MPR, multiplanar reformation; PCI, percutaneous coronary intervention, PDA, posterior descending artery, PL, posterolateral, RCA, right coronary artery, TAVR, transcatheter aortic valve replacement.

Pre-procedural planning was performed using the 3mensio (Pie Medical Imaging) software. Manual vessel tracing in curved multiplanar reconstruction was performed using either the coronary or mitral/tricuspid package. Despite the absence of contrast in the occluded segment, CTOs can be easily traced on coronary CCTA.^4^ Curved multiplanar reconstructions aid in identifying the proximal cap, plaque morphology (blunt vs tapered cap), the extent of calcifications, approximate CTO length, and distal cap location. These features are crucial for estimating whether the CTO can be crossed with antegrade wire escalation or if the wire will end up extra-plaque, leading to an antegrade dissection re-entry approach. The analysis also helped identify the contour of the entire vessel and the presence of tortuosity. Finally, 3-dimensional computed tomography (CT) reconstructions facilitated the precise visualization of the CTO trajectory and assisted in selecting the most optimal angiographic projection for orthogonal visualization and the directionality of wiring in the occluded segment. In long lesions, a side view of each CTO segment can be identified to eliminate foreshortening.

In case 1, the proximal cap was located with Gladius Mongo (Asahi Intecc) by selecting prespecified CT angles on fluoroscopy and performing intravascular ultrasound pullback from the diagonal branch into the left main coronary artery. The cap was punctured and the CTO segment was navigated with the Gaia3 (Asahi Intecc), crossing the lesion into true lumen. In case 2, CCTA was used to delineate the trajectory of the anomalous left circumflex artery and its proximity to the annulus and transcatheter aortic valve replacement. This was done due to concerns that the transcatheter aortic valve could have potentially compressed the lumen of the anomalous coronary artery during deployment. Predetermined CT angles were used to position the fluoroscopy camera at the most optimal angle for crossing the CTO. The proximal cap was engaged with the Pilot 200 (Abbott), which ended up extraplaque within the body of the CTO. Subsequently, the Gaia3 was redirected into the true lumen with a parallel wiring technique. In case 3, the proximal RCA CTO was crossed using a reverse controlled antegrade and retrograde tracking technique. The proximal cap was punctured with the Confianza Pro12 (Asahi Intecc). Following this, an antegrade knuckle was created with the Gladius Mongo; the Suoh3 (Asahi Intecc) crossed retrograde through the septal collateral, and Pilot 200 was knuckled retrograde. Finally, re-entry was attained with Gaia3 via reverse controlled antegrade and retrograde tracking; the distal right posterolateral CTO was crossed antegrade using Pilot 200 wire.

In challenging CTO cases with high J-CTO scores, CCTA highlights all abovementioned challenges and could aid in procedural success.

## Supplementary material

Supplemental Video 1 LAD CTO angiograms

Supplemental Video 2 LCx CTO angiograms

Supplemental Video 3 RCA CTO angiograms
